# Hemorrhages and risk factors in patients undergoing thromboprophylaxis in a respiratory critical care unit: a secondary data analysis of a cohort study

**DOI:** 10.1186/s40560-024-00756-w

**Published:** 2024-10-29

**Authors:** Wen‐Rui Lyu, Xiao Tang, Yu Jin, Rui Wang, Xu‐Yan Li, Ying Li, Chun‐Yan Zhang, Wei Zhao, Zhao‐Hui Tong, Bing Sun

**Affiliations:** 1 Department of Respiratory and Critical Care Medicine, Beijing Institute of Respiratory Medicine and Beijing Chao-Yang Hospital, Capital Medical University, 8 Gongren Tiyuchang Nanlu, Chaoyang District, Beijing, 100020 China; 2 Department of Ultrasonic Diagnosis, Beijing Institute of Respiratory Medicine and Beijing Chao-Yang Hospital, Capital Medical University, 8 Gongren Tiyuchang Nanlu, Chaoyang District, Beijing, 100020 China

**Keywords:** Thrombosis, Critical care, Risk factors, Anticoagulants, Risk assessment

## Abstract

**Objective:**

To verify whether the bleeding risk assessment guidelines from the 9th American College of Chest Physicians (ACCP) are prognostic for respiratory intensive care unit (RICU) patients and to explore risk factors for hemorrhages, we conducted a secondary data analysis based on our previously published cohort study of venous thromboembolism.

**Patients and methods:**

We performed a secondary data analysis on the single-center prospective cohort from our previous study. Patients admitted to the RICU at Beijing Chao-Yang Hospital from August 1, 2014 to December 31, 2020 were included and followed up until discharge.

**Results:**

The study enrolled 931 patients, of which 715 (76.8%) were at high risk of bleeding, while the remaining were at low risk. Of the total, 9.2% (86/931) suffered major bleeding, and no significant difference was found between the two risk groups (p = 0.601). High-risk patients had poor outcomes, including higher mortality and longer stays. Independent risk factors for major bleeding were APACHE II score ≥ 15; invasive pulmonary aspergillosis; therapeutic dose of anticoagulants; extracorporeal membrane oxygenation; and continuous renal replacement therapy. Blood transfusion not related to bleeding appeared to be an independent protective factor for major bleeding (OR 0.099, 95% CI 0.045–0.218, p < 0.001).

**Conclusion:**

Bleeding risk assessment models from the 9th ACCP guidelines may not be suitable for patients in RICU. Building a bleeding risk assessment model that is suitable for patients in all RICUs remains a challenge.

*Trial registration* ClinicalTrials.gov: NCT02213978.

## Introduction

Bleeding is a common complication in intensive care units (ICUs), with incidence varying from 1.4% to 10.9%[1–4]. Patients who experience major bleeding have been reported to have a higher incidence of mortality than those who do not (39.4% vs. 14.3%)[5]. Critically ill patients often have many conditions that increase the risk of bleeding. Several bleeding risk models have been developed to evaluate bleeding risk for both inpatients and outpatients [6–9] However, none of all models have been verified as suitable for ICU patients.

Patients in ICUs are usually considered at high risk for venous thrombus embolism (VTE) and need preventive measures. However, achieving optimal balance between the risks of bleeding and clotting is challenging [10]. The 9th American College of Chest Physicians (ACCP) for VTE prophylaxis propose a bleeding risk evaluation method, but its suitability for patients in ICUs remains unclear, and there is little consensus on risk factors for hemorrhages. We previously performed a prospective single-center cohort study of VTE in a respiratory intensive care unit (RICU) and found the thromboprophylaxis protocol, guided by 9th ACCP guidelines, to be effective. Most patients admitted to the RICU had mainly medical or respiratory conditions, with over 65% having pneumonia, more than 30% presenting with ARDS, and up to 65% requiring invasive mechanical ventilation. In addition, over 10% of patients received VV-ECMO treatment [11]. Building on this preliminary study, we performed a secondary analysis to verify the prognostic value of the risk assessment from the 9th ACCP guidelines and to explore the risk factors for major bleeding in RICU patients.

## Materials and methods

We performed a secondary data analysis on the single-center prospective cohort in our previous study [11]. Patients admitted to the RICU in Beijing Chao-Yang Hospital from August 1, 2014 to December 31, 2020 were included in this study and were followed up with until they were discharged. Attending physicians were interviewed daily to determine whether outcome events had occurred.

### Patients

Patients older than 18 years and were expected to stay in the ICU for more than 48 h were included. Exclusion criteria were as follows: bleeding occurring 3 months before admission, active gastroduodenal ulcer, and repeated admissions during the study progress. Our previous study of VTE in the RICU was reviewed and approved by the Ethics Committee of Beijing Chao-Yang Hospital (2014-Ke-142). Informed consent was obtained from the patients themselves or their legal guardians.

### Bleeding risk evaluation and VTE prophylaxis.

According to the 9^th^ ACCP guidelines, patients were ranked and divided into high- or low-risk bleeding, with evaluation completed within 48 h of admission. The evaluation criteria included factors such as age, gender, renal function, and recent hemorrhages. Patients received VTE prophylaxis according to both VTE risk and bleeding risk, with a standard drug prophylaxis consisting of 40 mg subcutaneous injection of enoxaparine per day. In some cases, such as for patients diagnosed with atrial fibrillation, chronic thromboembolic pulmonary hypertension, or post-cardiac surgery, the anticoagulant dosage was adjusted to a therapeutic level. Therapeutic doses were typically defined as Enoxaparine 10 mg/ 10 kg administered twice per day, fondaparinux 5 mg/7.5 mg per day, continuous intravenous infusion of unfractionated heparin (UFH) aimed at 1.5 times the baseline activated partial thrombin time (APTT), or warfarin with an international normalized ratio (INR) target between 2 and 3.

### Data collected

Patients’ characteristics, laboratory tests, and treatments were collected using case report forms, including demographic characteristics (age and gender), diagnosis, comorbidities, complications. Invasive operations performed within the first 48 h of admission were documented, including extracorporeal membrane oxygenation (ECMO) and continuous renal replacement therapy (CCRT). Blood transfusions not related to hemorrhages, such as transfusions of plasma, human fibrinogen, or platelet transfusions for patients with coagulation disorders, hypofibrinogenemia, or thrombocytopenia, were also recorded.

### Outcomes

#### Primary outcomes

Hemorrhages, evaluated and confirmed by attending physicians, were the primary outcome. They were grouped into two categories: major and non-major bleeding. Major bleeding, as defined by the Subcommittee of the International Society on Thrombosis and Haemostasis recommendations for non-surgical patients [12]. Non-major bleeding refers to clinically significant bleeding not matching the criteria of major bleeding, which includes gastrointestinal bleeding, urinary tract bleeding, oral or nasal bleeding, lower respiratory tract bleeding, retroperitoneal bleeding, skin bleeding, intracranial bleeding, surgical incision bleeding, and vaginal bleeding. All patients were observed until they either died or were discharged from the RICU.

#### Secondary outcome (a composite outcome)

Patients who died in the RICU or stayed more than 28 days were counted as the secondary outcome. Mortality was defined as death from any cause, and patients who gave up treatment during their RICU stay were also counted as dead.

### Statistical analysis

Case report forms were designed for data collection. Statistical analysis was performed with IBM SPSS 26.0. Categorical variables were described as frequency (percentage), and differences between groups were tested by the Chi-square test and Fisher's exact test. Continuous variables were described by median (interquartile range (IQR)). Differences between groups were tested by the U test. Some of the continuous variables were split into two levels based on median or apparent clinical significance cutoff. Multivariable logistics regression was used to modify confounding factors. Odds ratios (ORs) and 95% confidence intervals (CIs) for univariates were obtained by Chi-square, and those of multivariates were obtained by multivariable logistics regression. Variates with *P*<*.1* were included in the multivariate analysis. Collinearity diagnoses were used to further select variables for multivariate regression analysis. A value of *P*<*.05* was considered statistically significant. Multiple imputations were used for variates with missing values less than 20%. Generalized linear models were performed to detect interaction effects between factors. Figures were plotted by IBM SPSS 26.0, ProcessOn (https://www.processon.com), and GraphPad Prism 9.

### Bias and sensitivity analysis

Our study was a cohort study with an unpaired design, so there was a selection bias among the study subjects. The baseline table shows the differences between the cohorts in detail. Outcome measures were obtained using the same methods and definitions for patients in both cohorts. Age, gender, Acute Physiology, Age, and Chronic Health Evaluation II (APACHE II) score, platelet count, and renal dysfunction were the main planned confounders. The accuracy of the results was assessed by calculating ORs and their 95% CIs. Sensitivity analyses of risk factor exploration were performed using different handling methods for missing data.

## Results

**Fig. 1:**
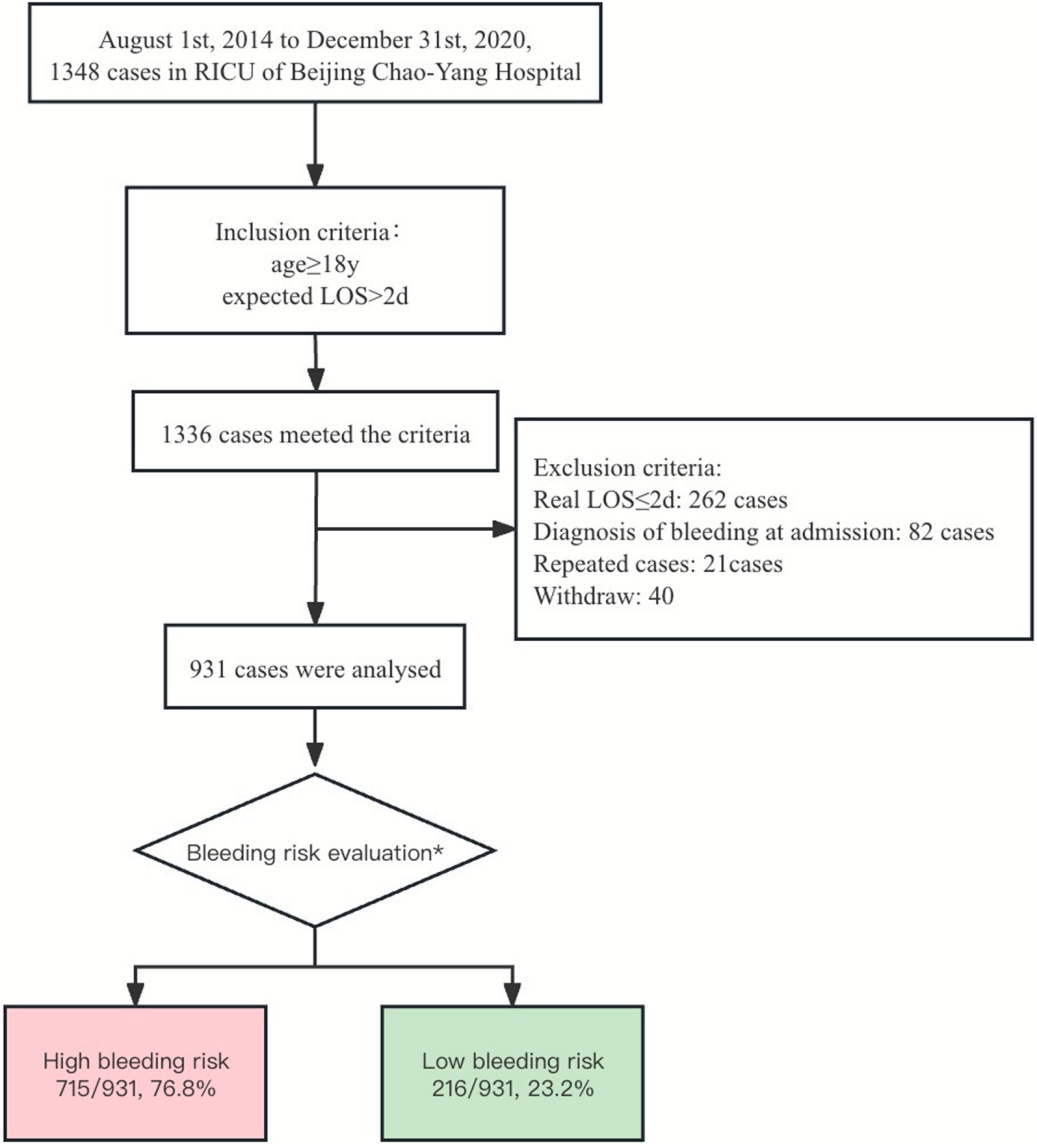
Study flow chart. *LOS* length of stay *Bleeding risk evaluation methods were conducted according to the 9th ACCP thromboprophylaxis guidelines^11^

Of 1,348 patients admitted to the RICU from August 1, 2014 to December 31, 2020, a total of 931 cases were ultimately enrolled in the study (Fig. 1).

### Patients’ characteristics

Of all patients, 715 (76.8%) were evaluated as being at high bleeding risk, while 216 (23.2%) were classified as low risk. For all patients admitted to the RICU, the median age was 61 years (IQR 47–70). Males constituted 66.3% of patients and the median APACHE II score was 15 (IQR 10–21). Patients were predominantly diagnosed at admission with pneumonia (67.9%) and acute respiratory distress syndrome (31.4%) (Table 1). The high-risk group had a higher proportion of males and patients with sepsis, malignant tumor, and acute renal insufficiency. In addition, patients in the high-risk group were also younger than those in the low-risk group.

**Table 1:** Patients’ characteristics

	All patients N = 931	Low bleeding risk N = 216	High bleeding risk N = 715	*p* value
Demographics				
Median age (IQR), year	61 (47, 70)	64 (51.3, 74)	61 (47, 70)	0.011
Age ≥ 65y, n (%)	377 (40.5)	105 (48.6)	272 (38.0)	0.006
Male, n (%)	617 (66.3)	27 (12.5)	590 (82.5)	< 0.001
Median body mass index (IOR), kg/m^2^	23.7 (20.8, 26.6)	23.4 (20.8, 27.2)	23.8 (20.8, 26.5)	0.919
Body mass index ≥ 24 kg/m^2^, n (%)	427 (46.7)	97 (45.5)	330 (47.0)	0.707
APACHE II score (IQR)	15(10, 21)	14 (10, 20)	15 (10, 22)	0.160
APACHE II score ≥ 15, n (%)	455 (52.2)	102 (49.5)	353 (53.0)	0.381
Caprini VTE risk score (IQR)	5 (3)	4 (3)	5 (4)	0.010
Diagnosis at admission, n (%)				
Pneumonia	629 (67.6)	135 (62.5)	494 (69.1)	0.070
Diffuse parenchymal lung disease	102 (11.0)	25 (11.6)	77 (10.8)	0.750
Acute exacerbations of chronic obstructive pulmonary disease	102 (11.0)	33 (15.3)	69 (9.7)	0.020
Acute exacerbations of asthma	18 (1.9)	8 (3.7)	10 (1.4)	0.061
Bronchiectasis	18 (1.9)	7 (3.2)	11 (1.5)	0.191
Invasive pulmonary aspergillosis	26 (2.8)	7 (3.2)	19 (2.7)	0.648
Sepsis	76 (8.2)	7 (3.2)	69 (9.7)	0.003
Acute respiratory distress syndrome	292 (31.4)	57 (26.4)	235 (32.9)	0.072
Acute renal disfunction	79 (8.5)	9 (4.2)	70 (9.8)	0.009
Acute liver disfunction	45 (4.8)	11 (5.1)	34 (4.8)	0.839
Comorbidities, n (%)				
Hypertension	376 (40.4)	93 (43.1)	283 (39.6)	0.362
Diabetes mellitus	208 (22.3)	46 (21.3)	162 (22.7)	0.674
Coronary artery disease	116 (12.5)	26 (12.0)	90 (12.6)	0.830
Chronic renal failure	84 (9.0)	14 (6.5)	70 (9.8)	0.137
Malignant tumor	92 (9.9)	11 (5.1)	81 (11.3)	0.007
After an organ transplantation	62 (6.7)	13 (6.0)	49 (6.9)	0.666
Liver cirrhosis or liver disfunction	49 (5.3)	9 (4.2)	40 (5.6)	0.410
Drug history, n (%)				
Long-term oral glucocorticoids	139 (14.9)	36 (16.7)	103 (14.4)	0.418
Aspirin or clopidogrel	33 (3.5)	8 (3.7)	25 (3.5)	0.885
Anticoagulants	52 (5.6)	16 (7.4)	36 (5.0)	0.183
Laboratory tests				
Hemoglobin (g/L)	110.0 (92.0, 128.0)	108.0 (93.0, 123.8)	111.0 (91.0, 131.0)	0.093
Platelet(*10^9^/L)	172.0 (116.5, 246.0)	184.0 (134.5, 262.0)	165.0 (114.0, 241.5)	0.003
Prothrombin time (second)	12.8 (11.7, 14.2)	12.3 (11.4, 13.4)	13.0 (11.9, 14.4)	< 0.001
Activated partial thromboplastin time (second)	31.0 (25.9, 37.8)	29.3 (24.9, 36.5)	31.5 (26.1, 38.5)	0.001
Fibrinogen (mg/L)	397.8 (280.2, 523.4)	394.1 (278.4, 484.8)	400.6 (281.4, 538.1)	0.257
Creatinine (μmol/L)	73.8 (51.7, 118.3)	58.9 (45.3, 84.2)	77.9 (54.4, 131.1)	< 0.001

*APACHE II score* Acute Physiology, Age, Chronic Health Evaluation II score, *IQR* interquartile range, *VTE* venous thrombus embolism

*p* < 0.05 was statistically different

Patients in the high-risk group had lower platelet levels and higher prothrombin times and APTT levels. Additionally, high-risk patients had higher levels of bilirubin, C-reactive protein, erythrocyte series resistance, and creatinine. However, no differences were found between the two groups in terms of hemoglobin, fibrinogen, or albumin.

During their RICU stay, patients in the high-risk group underwent more procedures such as tracheostomy (154/715 vs. 30/216, *P* = 0*.013*), CRRT (160/715 vs. 17/216, *P* <0*.001*), ECMO (*P* <0*.001*), and intravascular catheter (511/715 vs. 121/216, *P* <0*.001*), as well as blood transfusions not related to bleeding (232/715 vs. 47/216, *P* =0*.003*) and proton pump inhibitors (532/715 vs. 133/216, *P* <0*.001*), than the low-risk group.

### Outcomes

Of all patients, 16.4% (153/931) experienced hemorrhages. This total included 14.4% (31/216) of low-risk patients and 17.1% (122/715) of high-risk patients (p= 0.346). Major bleeding occurred in 9.2% (86/931) of all patients, including 8.3% (18/216) of low-risk patients and 9.5% (68/715) of high-risk patients (*p* = *0*.601). Differences between the two groups did not change after adjustment for age (≥65 years), gender, AECOPD, sepsis, renal dysfunction, platelet count <50×10^9^/L, malignant tumor, and APACHE II score ≥15. Gastrointestinal bleeding was the most common site of bleeding, accounting for more than half of all hemorrhages cases. Details are shown in Table 2. The length of stay in the RICU was 12 d (IQR 7, 20).

**Table 2:** Outcomes

	All Patients N = 931	Low bleeding risk N = 216	High bleeding risk N = 715	p value^*^	Patients with major bleeding N = 86	*p* value^$^	Patients with hemorrhages N = 153	p value^&^
Primary outcomes								
Major bleeding	86 (9.2)	18 (8.3)	68 (9.5)	0.601	–	–	–	–
Hemorrhages	153 (16.4)	31 (14.4)	122 (17.1)	0.346				
Secondary outcomes								
Died in RICU or length of stay in RICU ≥ 28d	381 (40.9)	70 (32.4)	311 (43.5)	0.004	72 (83.7)	** < 0.001**	116 (75.8)	< 0.001
Died in RICU	270 (29.0)	50 (23.1)	220 (30.8)	0.031	53 (61.6)	** < 0.001**	85 (55.6)	< 0.001
Length of stay in RICU ≥ 28d, n (%)	151 (16.2)	25 (11.6)	126 (17.6)	0.035	34 (39.5)	** < 0.001**	53 (34.6)	< 0.001

*APACHE II score* Acute Physiology, Age, Chronic Health Evaluation II score, *RICU* respiratory intensive care unit

^*^Confounding factors were adjusted by logistic regression analysis respectively. The difference in mortality between the two cohorts still existed after being adjusted by age (≥ 65y), gender, acute exacerbations of chronic obstructive pulmonary disease, sepsis, renal dysfunction, platelet (< 50*10^9^/L), malignant tumor, and APACHE II score (≥ 15)

^&^Comparison between patients with and without hemorrhages

^$^Comparison between patients with and without major bleeding

*p* < 0.05 was statistically different

Secondary outcomes applied to 40.9% (381/931) of all patients. This number included 32.4% (70/216) of low-risk patients and 43.5% (311/715) of high-risk patients (p =0.004). Differences between the two groups still existed after adjustment for age (≥65 years), AECOPD, sepsis, acute renal dysfunction, malignant tumor, and APACHE II score ≥15. Patients who also experienced bleeding or major bleeding had poorer outcomes than those who did not, with a higher incidence of death or longer stays in the RICU for more than 28 days (*P*<0.001 for both).

### Risk factors for major bleeding

Univariate analysis involved 17 factors, including APACHE II score ≥15, invasive pulmonary aspergillosis, acute renal dysfunction, acute liver dysfunction, white blood cells ≥9.5×10^9^/L, aspartate transaminase ≥40 U/L, direct bilirubin ≥6.8 μmol/L, and mechanical ventilation.

**Fig. 2:**
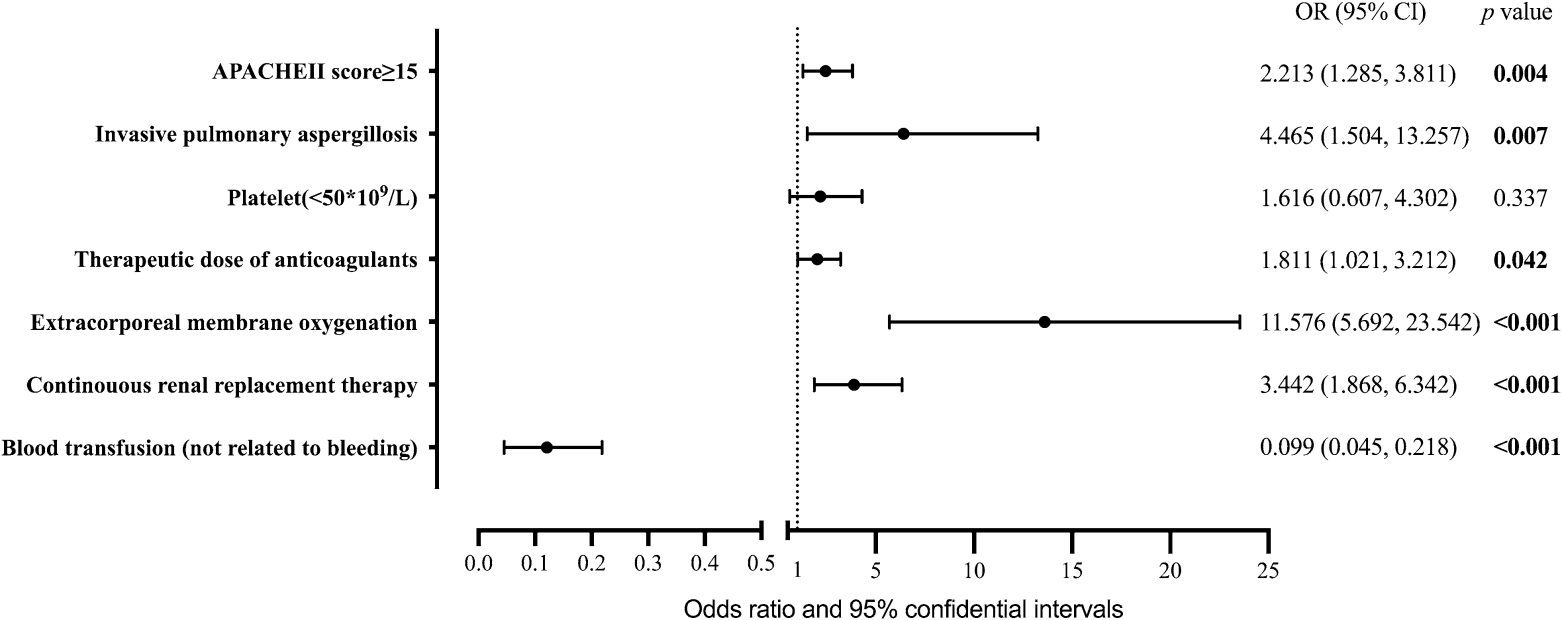
Forest plot of multivariate regression of major bleeding. *APACHE II score* Acute Physiology, Age, and Chronic Health Evaluation II score, *OR* odds ratio, *CI* confidence interval

Independent risk factors for major bleeding included APACHE II score ≥15 (OR 2.213, 95% CI 1.285–3.811, *P* =0*.004*), invasive pulmonary aspergillosis (OR 4.465, 95% CI 1.504–13.257, *P* =0*.007*), therapeutic dose of anticoagulants (OR 1.811, 95% CI 1.021–3.212, *P* =0*.042*), ECMO (OR 11.576, 95% CI 5.692–23.542, *P* <0*.001*), and CRRT (OR 3.442, 95% CI 1.868–6.342, *P* <0*.001*). Blood transfusion not related to bleeding appeared to be an independent protective factor against major bleeding (OR 0.099, 95% CI 0.045–0.218, *P* < *0.001*) (Figure 2). This conclusion was largely consistent with the raw data, except for the therapeutic dose of coagulants. In the unimputed data, a therapeutic dose of coagulants treatment was not an independent risk factor for major bleeding (OR 1.767, 95% CI 0.979, 3.189, *P* = *0.059*).

We found several interaction effects: CRRT with blood transfusion not related to bleeding (*P* = *0.001*), ECMO with platelets (*P* = *0.048*), ECMO with blood transfusion not related to bleeding (*P* = *0.007*), and APACHE II score with a therapeutic dose of anticoagulants (*P* = *0.046*). Stratified analysis revealed that the protective effect of blood transfusion not related to bleeding was more significant for patients who were treated with CRRT than for patients who were not treated and was more significant for patients treated with ECMO than for those who were not treated. In non-ECMO patients, lower platelet levels remained an independent risk factor for major bleeding; however, in ECMO patients, platelet levels were not associated with hemorrhages. Therapeutic dose of anticoagulant was an independent risk factor for patients with an APACHE II score less than 15.

## Discussion

This study was designed to explore the incidence of hemorrhages under a VTE prophylaxis protocol [11]. Our study found that major bleeding occurred in 9.2% of the total cohort. According to the projected model from the 9th ACCP guideline, more than half of the critically ill patients had high risk of bleeding. However, there was no significant difference in the incidence of hemorrhages between the high-risk group and low-risk group as stratified by the guidelines. The severity of critical conditions, presence of invasive pulmonary aspergillus, therapeutic dose of anticoagulants, ECMO, and CRRT were independent risk factors for major bleeding.

There are several reasons that the model was not applicable to the patients in this cohort. First, a higher proportion of patients in our cohort received ECMO (12.6%, 117/931) and mechanical ventilation (85.4%, 795/931) compared to those in other studies, which may lead to a higher bleeding rate, as previous studies have confirmed that these are risk factors. However, in a prospective study, investigators may be more sensitive to identifying clinical outcomes, which may lead to a relatively higher recording of incidence. Second, bleeding risks were evaluated before VTE prophylaxis and were given ongoing attention after initial assessment. Strengthened monitoring of bleeding risk during the treatment process led to the prevention of some hemorrhages in patients at high risk. As described above, prophylactic methods included more blood transfusions for non-hemorrhagic patients and the use of proton pump inhibitors. Overall, we conclude that timely development of new bleeding risk assessment systems for patients in RICUs is still necessary.

Researchers focusing on hemorrhages in ICUs have found that coagulopathy, low platelet count, renal failure, tracheotomy, and mechanical ventilation are primary risk factors for clinically significant hemorrhages [13–15]. However, due to differences in disease severity, patient characteristics, and anticoagulation management across different types of ICUs, most of these results had low certainty [16]. Thus, no consensus has been reached on the risk factors for hemorrhages in patients in ICUs. Alexander et al. used deep learning methods to predict several complications (including bleeding) in cardiac ICUs in real-time [17], which appeared to offer a promising avenue for risk assessment. However, this method has faced criticism regarding its accuracy, data quality, and utility [18]. As expected, building a universal prediction model is difficult; however, identifying some preventable risk factors remains meaningful. In this study, we found several independent risk factors, as mentioned above.

Patients on ECMO were at risk for both VTE and hemorrhages. Our previously published findings confirmed that ECMO was an independent risk factor of VTE in RICUs [11]. In this data analysis, ECMO also emerged as an independent risk factor for hemorrhages. Additionally, our study showed that a platelet count of less than 50 × 10^9^/L was not an independent risk factor for bleeding in ECMO patients but was harmful for non-ECMO patients. Reportedly, the bleeding rate for patients in ECMO reached up to 45% [19], and a platelet count higher than 100 × 10^9^/L was considered a protective factor [20]. While many studies have shown a relationship between ECMO and thrombocytopenia [21], the relationship between thrombocytopenia and hemorrhages in ECMO patients remains unclear. A multicenter retrospective study showed that platelet count and fibrinogen level were risk factors for intracranial hemorrhage for V-A ECMO patients [22]. However, some recently reported studies indicated that platelet count was not associated with hemorrhages for venous-venous (V-V) ECMO patients [23, 24]. Our study did not separately analyze V-A versus V-V ECMO.

Renal dysfunction is regarded as a risk factor for bleeding [15, 25], and our current study showed that CRRT (due to renal dysfunction) was an independent risk factor for bleeding. Our study also found an interaction effect between CRRT/ECMO and protective transfusion, implying that the protective effect of blood transfusion was more pronounced in patients undergoing CRRT or ECMO. At present, a strict transfusion strategy is generally adopted worldwide. However, guidelines for transfusion in ICUs are limited, and there remains debate regarding the benefits of prophylactic transfusion [26, 27]. We speculate that for patients at high risk of bleeding, such as those undergoing CRRT or ECMO, protective transfusion can reduce the incidence of major bleeding. This warrants further research.

One possible reason for the predisposition to hemorrhage associated with aspergillus is that this infection in the bloodstream causes vascular damage and increases bleeding. An in vitro study found that aspergillosis induced local hemolysis in infected tissues [28]. Furthermore, aspergillus produces elastase, which damages local tissues and can cause localized bleeding [29]. Antifungal azole drugs can interact indirectly with multiple drugs, which warrants attention. Unlike previously reported cases of local infection site bleeding, our study found that the main bleeding site for patients diagnosed with aspergillus pneumonia was gastrointestinal (Supplementary Table 5), rather than at the infection site. However, no findings have established a direct relationship between aspergillosis and gastrointestinal bleeding. Antacids are considered to have protective effects for patients at high risk of bleeding. However, in our study, antacids such as proton pump inhibitor (PPI) and H2-receptor antagonist (H2RA) did not show a protective effect. Some research results show that PPI, H2RA, and sucralose can prevent stress ulcers [30], yet the preventive effect of PPIs on gastrointestinal bleeding remains controversial [31]. Many centers are currently evaluating the effectiveness and safety of PPIs compared with H2RA in preventing stress ulcers [32]. Finally, while being male has been considered a risk factor for hemorrhages in acutely ill patients [33], we did not find it to be an independent risk factor in our study.

There are several limitations to our study. First, as a single-center study conducted in a RICU, it is subjected to unavoidable selection bias. Second, this study lacked data on the timing of hemorrhages and deaths, and the study’s follow-up period was short; therefore, 60-day and 90-day survival rates were not provided. Third, in clinical practice, the risks of bleeding are evaluated in real-time. However, our study was limited to analyzing the association between hemorrhages and bleeding risk as evaluated at admission. Furthermore, as this study involved a secondary data analysis, the determination of sample size was not repeated. Although patients at high bleeding risk exhibited a higher incidence of bleeding compared to those at low risk, the lack of statistical significance may be attributed to an insufficient sample size.

## Conclusion

Current bleeding risk assessment models may not be suitable for critically ill patients in RICUs, who are at high risk for both thrombosis and bleeding. Still, it is challenging to build a bleeding risk assessment model for such patients.

## Data Availability

The datasets analyzed during the current study are available from the corresponding author upon reasonable request.

## Abbreviations

ACCP: American college of chest physicians
AECOPD: Acute exacerbation of chronic obstructive pulmonary disease
APACHE II score: Acute physiology and chronic health evaluation score
CI: Confidence intervals
CRRT: Continuous renal replacement therapy
ECMO: Extracorporeal membrane oxygenation
H2RA: H2-receptor antagonist
ICU: Intensive care unit
IQR: Interquartile range
OR: Odds ratio
RICU: Respiratory ICU
PPI: Proton pump inhibitor
VTE: Venous thromboembolism
